# Detection of foreign bodies in the canine stomach using capsule endoscopy: a randomized trial

**DOI:** 10.3389/fvets.2024.1440831

**Published:** 2024-08-07

**Authors:** Ui-Yeon Kim, Young Joo Kim, Joon Woo Lee, Munso Kim, Hyomi Jang, Dong-In Jung

**Affiliations:** ^1^Institute of Animal Medicine, College of Veterinary Medicine, Gyeongsang National University, Jinju, Republic of Korea; ^2^College of Veterinary Medicine, Western University of Health Sciences, Pomona, CA, United States; ^3^VIP Animal Medical Center, Seoul, Republic of Korea

**Keywords:** capsule endoscopy, dog, foreign body, stomach, vomiting

## Abstract

**Introduction:**

This study aimed to assess the effectiveness of capsule endoscopy in detecting gastric foreign bodies in normal dogs, considering variations in the number of foreign bodies and the gastric environment.

**Methods:**

Five healthy male beagles were administered virtual, non-harmful foreign objects that maintained their shape in the stomach. Capsule endoscopy was performed and the images were evaluated by veterinarians and non-veterinarians.

**Results:**

The overall sensitivity and specificity of capsule endoscopy were 99.1 and 90.4%, respectively. Sensitivity and specificity were comparable between veterinarians and non-veterinarians. Sensitivity and specificity in the veterinarian group were 98.7 and 91.2%, respectively, whereas those in the non-veterinarian group were 100 and 88.5%, respectively.

**Discussion:**

Capsule endoscopy is a valuable alternative diagnostic tool for identifying foreign bodies in the stomach, particularly in challenging cases in which conventional imaging or invasive approaches have limitations.

## 1 Introduction

Capsule endoscopy is a non-invasive procedure that utilizes an orally introduced capsule equipped with a camera to capture images of the gastrointestinal tract, enabling visual examination and potential diagnosis (1, 2). This technique overcomes the limitations associated with conventional endoscopy such as difficulties in visualizing the distal small intestine. One of the critical advantages of capsule endoscopy is that it does not require general anesthesia or sedation, and the captured images do not require intensive manipulation required by traditional endoscopic procedures (3). Capsule endoscopy is widely used in human medicine to diagnose gastrointestinal conditions including obscure gastrointestinal bleeding, Crohn’s disease, and tumors (4–7). In veterinary medicine, it has been used to evaluate the effectiveness of anthelmintics, assess gastrointestinal transit time, and detect abnormal mucosal lesions in cases of suspected gastrointestinal bleeding (8–12).

Dogs, especially young and medium-to-large-sized breeds, are at high risk of foreign body ingestion (13, 14). The clinical signs of foreign body ingestion in dogs include vomiting, lethargy, appetite loss, and abdominal pain. Partial or complete obstruction of the gastrointestinal tract caused by foreign bodies can induce acid–base imbalance, electrolyte imbalance, hypovolemia, toxemia, and tissue necrosis (13, 15). Prompt diagnosis and treatment are crucial in these cases. Currently, imaging evaluation techniques such as radiography, ultrasonography, and barium contrast radiography are commonly used to detect foreign bodies. Alternatively, more invasive approaches, such as gastrointestinal endoscopy or exploratory laparotomy, may be necessary. However, there are instances where foreign bodies cannot be detected through imaging evaluation or invasive procedures may pose challenges or risks based on the patient’s condition (14, 16–20). In such scenarios, capsule endoscopy may offer an optimal solution, enabling direct gastrointestinal tract examination without sedation or anesthesia.

Therefore, this study aimed to assess the efficacy capsule endoscopy in detecting foreign bodies in varying quantities in the stomach of healthy dogs.

## 2 Materials and methods

### 2.1 Animal preparation

This study enrolled five adult male beagles, aged approximately 3 years and weighing 9.3–14.4 kg (mean, 10.9 kg). All dogs were of the same breed and had comparable age and body weight (Table 1). Each dog was individually housed in a cage and regularly fed commercial dry dog food. Before commencing capsule endoscopy, all dogs were physically examined, which revealed no specific concerns. For the 9 months leading up to the study, none of the dogs had received any medication or exhibited any digestive tract symptoms. Based on these observations and their medical histories, the participants were deemed clinically healthy. All procedures involving dogs complied with the guidelines approved by the Institutional Animal Care and Use Committee (IACUC) of Gyeongsang National University (GNU-190827-D0041).

**Table 1:** Baseline characteristics of dogs in this study.

Number of dogs	Breed	Sex	Age (years)	BW (kg)
No. 1	Beagle	M	3	14.4
No. 2	Beagle	M	3	11.0
No. 3	Beagle	M	3	9.4
No. 4	Beagle	M	3	10.8
No. 5	Beagle	M	3	9.3

M, male.

### 2.2 Capsule endoscopy procedure

**Figure 1:**
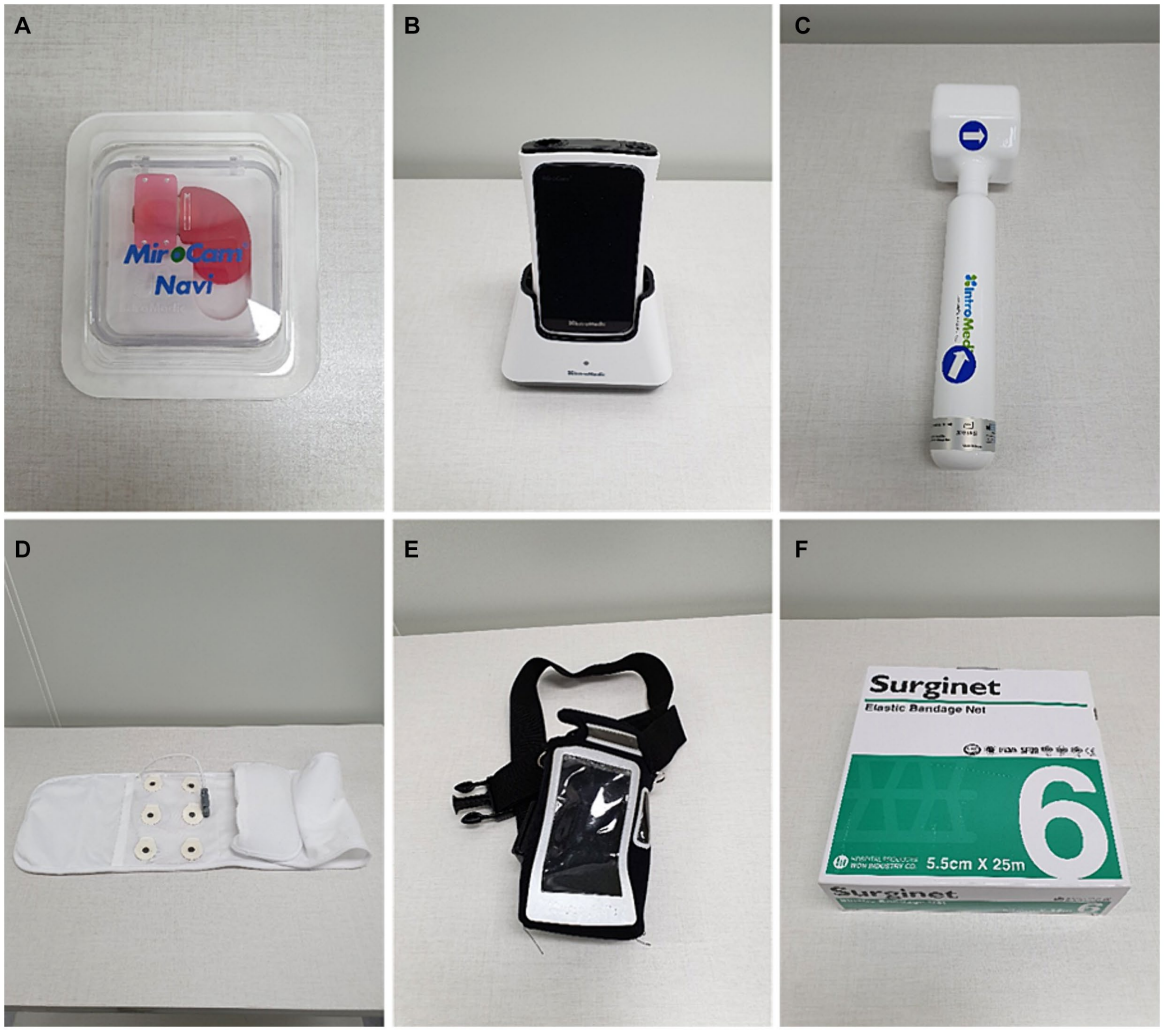
Capsule endoscopy equipment used in this study. **(A)** Capsule endoscope (MiroCam^®^ MC-1200M). **(B)** Receiver and receiver cradle (MiroCam^®^ MR2000). **(C)** Hand-held magnet. **(D)** Data belt. **(E)** Receiver pouch. **(F)** Elastic bandage net.

The capsule endoscopy system used in this study comprised a wireless endoscopic capsule (MiroCam^®^ Capsule MC-1200M, Intromedic, Seoul, Republic of Korea), a receiver (MiroCam^®^ Receiver MR2000, Intromedic, Seoul, Republic of Korea) for image transmission from the capsule, a software program (MiroViewTM 4.0 software, Intromedic, Seoul, Republic of Korea) for image analysis, and assorted accessories. The capsule measured 25.5 × 11 mm and weighed 4.2 g. It featured a miniature camera with a 170° field of view and the capability to operate for up to 8 h with six white light sources, a transfer device, and a battery. The images captured by the capsule were transmitted to a receiver and analyzed using a software program on a computer workstation. The uploaded images can be edited, played, paused, and manipulated at various speeds using this software. “Real-time” images can also be viewed through the receiver’s screen, enabling the assessment of the receiver’s status (e.g., procedure duration, signal strength, and remaining battery). The accessories included a data belt to connect the receiver, handheld magnet to facilitate capsule movement and rotation, receiver pouch, and elastic bandage net (Figure 1).

**Figure 2:**
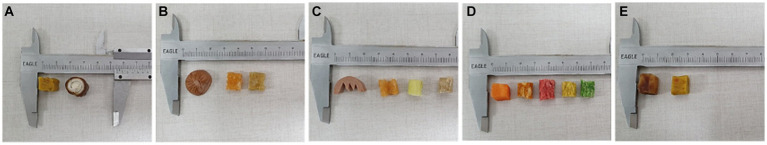
Foreign bodies administered to each dog in this study. **(A)** Two foreign bodies (treats) administered to Dog No. 1. **(B)** Three foreign bodies (sausage and treats) administered to Dog No. 2. **(C)** Four foreign bodies (sausage and treats) administered to Dog No. 3. **(D)** Five foreign bodies (carrot and treats) administered to Dog No. 4. **(E)** Two foreign bodies (treats) administered to Dog No. 5.

Given the inappropriateness of introducing actual foreign bodies typically ingested by dogs in clinical settings, we created a virtual foreign body model that posed no harm to dogs and remained undigested during examination. Our virtual foreign bodies comprised dog treats, sausage pieces, and carrot pieces, each measuring 12 × 12 cm. Different foreign bodies were administered individually to each dog (Figure 2).

In this study, we assumed that foreign bodies were radiolucent and undetectable on abdominal radiographs. Abdominal radiography using a Regius model 190 in the dorsal and right lateral views was conducted before and immediately after foreign body administration, and images were evaluated using the Digital Imaging and Communications in Medicine workstation. No gastric foreign bodies were detected during radiographic evaluation.

**Figure 3:**
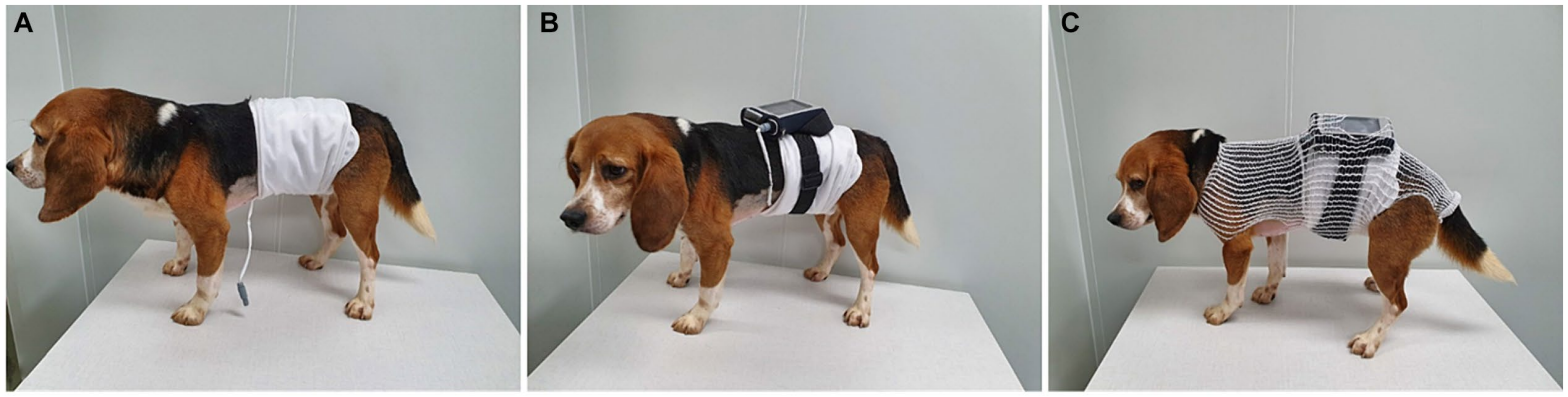
Capsule endoscopy equipment fixed on the dog’s body. **(A)** Data belt attached to the abdomen. **(B)** Receiver fixed to the back and connected to the data belt. **(C)** Equipment covered by elastic bandage net.

The capsules were administered 15 min after foreign body administration. Each dog underwent tests at intervals exceeding 1 month, and all dogs were fasted for >12 h before the examination. Prior to capsule administration, the electrode was attached to the dog’s abdomen, the data belt was fastened around the abdomen, and the receiver was placed in a pouch and secured to the dog’s back before connecting it to the data belt. The apparatus was secured by encasing the body in an elastic bandage net (Figure 3). Foreign bodies and capsules were administered, and images were transmitted from the capsule and observed on the receiver’s screen. A handheld magnet was used to steer the capsule for easy detection of foreign bodies. During capsule endoscopy, the dogs were permitted normal behavior, excluding eating and drinking. Capsule endoscopy times ranged from 56 min to 6 h 29 min, with some capsules reaching the small or large intestine. After endoscopy, the receiver was connected to the workstation and images were extracted for interpretation using a software program on the computer workstation. All the capsules were eventually excreted in the feces without gastric retention.

### 2.3 Group settings

Group 1 consisted of dogs administered only the capsule (without foreign bodies), whereas Group 2 included dogs administered the capsule along with foreign bodies. In Group 2, the number of foreign bodies varied from Dog No. 1 to Dog No. 4. For Dog No. 5, the number of foreign bodies was the same as that for Dog No. 1; however, additional food was administered (Table 2). Three comparisons were performed in this study.

**Table 2:** Group settings.

Number of dogs	Number of foreign bodies	
	Group 1	Group 2
No. 1	0	2
No. 2	0	3
No. 3	0	4
No. 4	0	5
No. 5	0	2 (+food)

Group 1, group of administration capsule only (without foreign body). Group 2, group of administration capsule with foreign body.

In the first comparison, the detection capability of capsule endoscopy was evaluated by comparing Group 1 (capsule only) with Group 2 (capsule with a foreign body).

A second comparison was conducted among four dogs (Dogs 1–4) in Group 2. The capsule endoscopy detection capability was assessed by varying the number of foreign bodies in dogs.

The third comparison was made between Dogs 1 and 5 (both dogs were administered with two foreign bodies). However, the detection capability of capsule endoscopy was assessed in two different scenarios: Dog No. 1 without additional food and Dog No. 5 with additional food. This was performed to determine the impact of gastric ingesta, introduced by the additional food administration in Dog No. 5, on the capability of capsule endoscopy to detect foreign bodies.

### 2.4 Capsule image interpretation and randomization

Ten capsule endoscopy images were acquired from Group 1 (capsule administration only) and Group 2 (capsule administration with foreign bodies). The transit time of a capsule endoscope from the stomach to the intestines varies from approximately 50 min to 5–6 h. To standardize the experimental conditions, the imaging data were normalized to a 50 min period based on the shortest observed gastric retention time. These images were independently interpreted by 16 veterinarians from the Gyeongsang National University Veterinary Medical Center and seven veterinary students from the College of Veterinary Medicine at Gyeongsang National University. All interpreters were blinded to the grouping of participants and the specific conditions under which the images were captured. Additionally, the order in which the images were presented to the interpreters was randomized and each interpreter was blinded to the findings of the others.

Interpretation values were calculated as percentages by converting the ratio of detected foreign bodies to the total number of foreign bodies administered to each individual. Sensitivity was defined as the number of results in which one or more capsules were detected divided by the total number of results in the group without foreign body administration and multiplied by 100. Specificity was defined as the number of results in which no foreign bodies were detected divided by the total number of results in the group without foreign body administration and multiplied by 100. Images of gastrointestinal tract regions other than the stomach were excluded during interpretation.

### 2.5 Statistical analysis

The Jonckheere–Terpstra test was used to determine the capsule endoscopy detection capability under different conditions, such as different numbers of foreign bodies. The Mann–Whitney *U* test was employed to compare the detection capability of capsule endoscopy when the gastric environment varied with the same number of foreign bodies. All analyses were conducted using SPSS statistical software (SPSS 25.0.0 for Windows, IBM, Armonk, NY, United States). Statistical significance was set at *p* < 0.05.

For calculating the intra-rater reliability within veterinarians and veterinary students, we utilized intra-class correlation coefficients (ICC). Given that each rater’s effect was random, we employed a two-way mixed effects ICC for absolute agreement. ICC agreement was represented with single measures and 95% confidence intervals (95% CI). Reliability interpretation was based on the following cut-off values: ICC <0.5 = poor, 0.5–0.75 = moderate, 0.75–0.9 = good, and >0.90 = excellent reliability.

To estimate the agreement of ratings between the veterinarian group and the veterinary student group, we presented a Bland–Altman plot with 95% limits of agreement. The Bland–Altman plot measured the difference between ratings using a scatterplot, with the *x*-axis representing the average of ratings for each group and the *y*-axis representing the differences between the two ratings. The mean difference and its 95% confidence limits were also plotted. Since our data consisted of integer rating values, jittering with random noise was utilized for representation. We employed the statistical software R, version 4.3.0, for calculating the intra-rater reliability analyses.

## 3 Results

### 3.1 Detection capability of capsule endoscopy in Group 1

**Figure 4:**
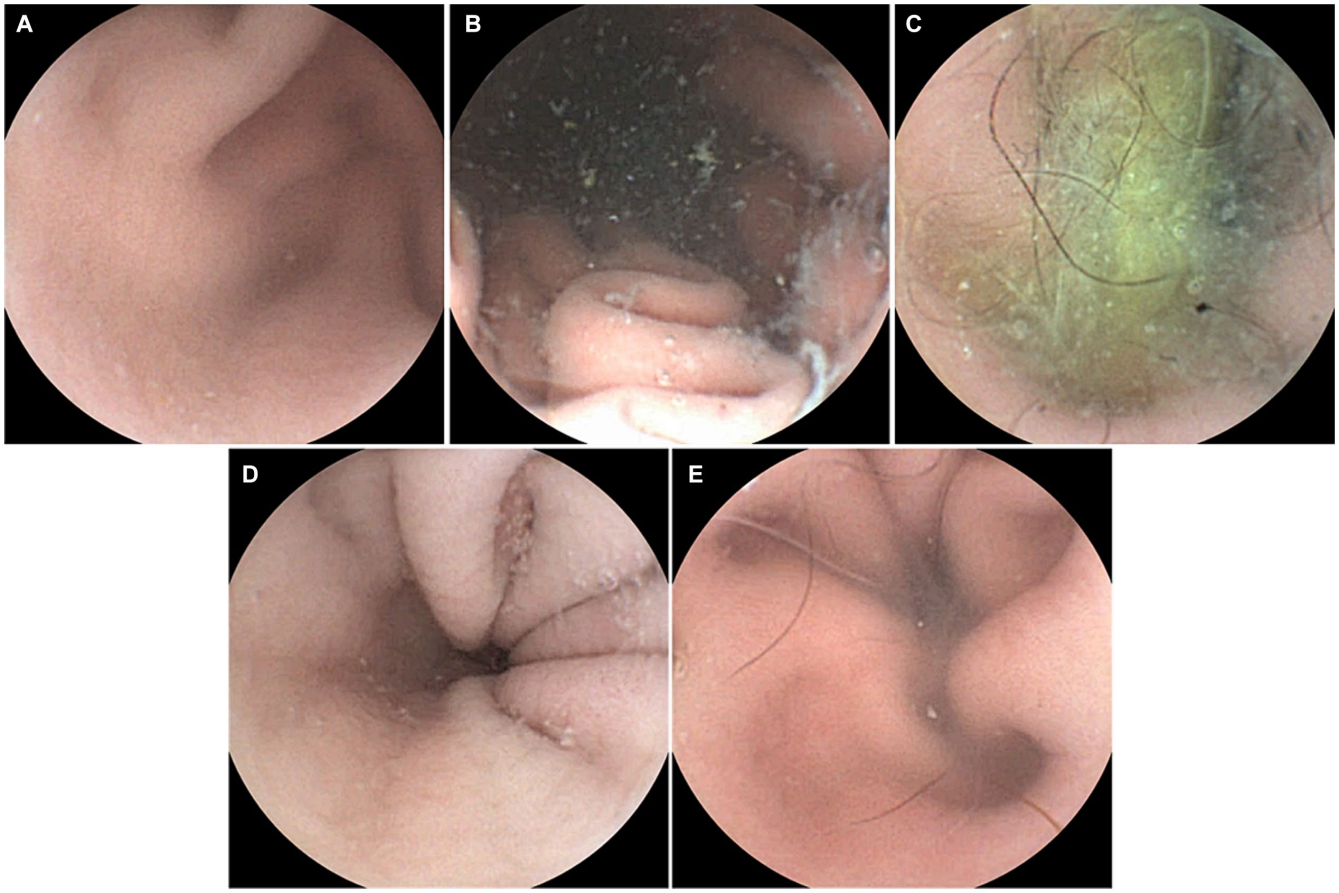
Capsule endoscopic images of Group 1 (administration of capsule endoscope only). **(A)** Dog No. 1. **(B)** Dog No. 2. **(C)** Dog No. 3. **(D)** Dog No. 4. **(E)** Dog No. 5.

The capsule endoscopic images of Group 1 are depicted in Figure 4. The interpreters’ interpretation values for Group 1 are listed in Table 3. Interpreters 1–16 were veterinarians and interpreters 17–23 were veterinary students. There was no significant difference in the specificity between the veterinarian and veterinary student groups (veterinarian group: 91.2% vs. veterinary student group: 88.5%; *p* = 0.624) (Table 4).

**Table 3:** Interpretation values of interpreters in Group 1.

Number of dogs (number of FB)	Number of detected FB																						
	Veterinarian																Veterinary student						
	1	2	3	4	5	6	7	8	9	10	11	12	13	14	15	16	17	18	19	20	21	22	23
No. 1 (0)	0	1	0	0	0	1	0	1	1	2	1	0	1	0	0	0	0	0	0	2	1	2	2
No. 2 (0)	0	0	0	0	0	0	0	0	0	0	0	0	0	0	0	0	0	0	0	0	0	0	0
No. 3 (0)	0	0	0	0	0	0	0	0	0	0	0	0	0	0	0	0	0	0	0	0	0	0	0
No. 4 (0)	0	0	0	0	0	0	0	0	0	0	0	0	0	0	0	0	0	0	0	0	0	0	0
No. 5 (0)	0	0	0	0	0	0	0	0	0	0	0	0	0	0	0	0	0	0	0	0	0	0	0

FB, foreign body.

**Table 4:** Total number of interpretation values of interpreters in Group 1.

	Total number of interpretation values	
	Veterinarians (*n* = 16)	Veterinary students (*n* = 7)
Number of interpretation values which one or more FB were detected	7	4
Number of interpretation values which FB were not detected	73	31
Specificity	91.2%	88.5%

FB, foreign body.

### 3.2 Detection capability of capsule endoscopy in Group 2

**Figure 5:**
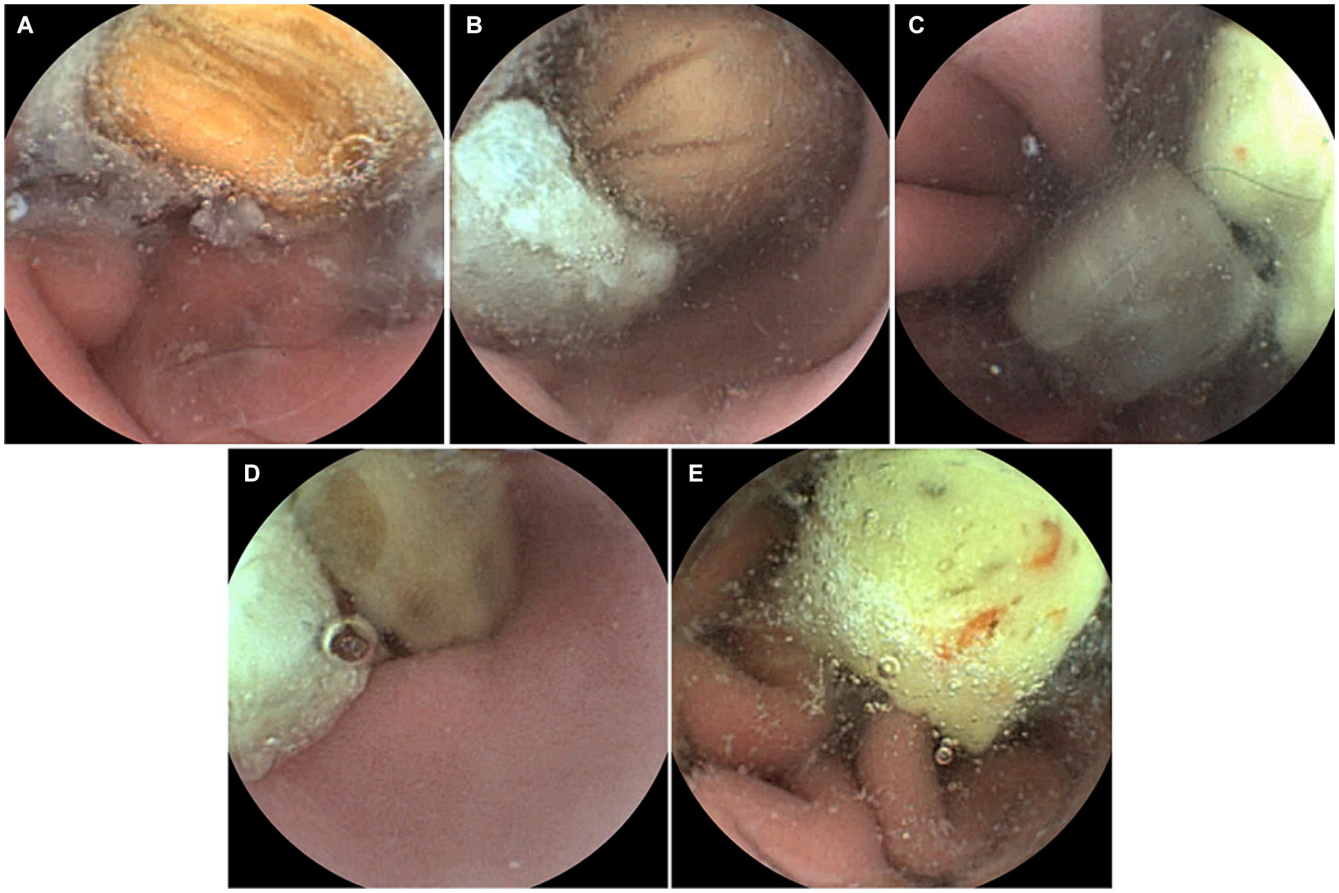
Capsule endoscopic images of Group 2 (administration of capsule with foreign body). **(A)** Dog No. 1 (administration of 2 foreign bodies). **(B)** Dog No. 2 (administration of 3 foreign bodies). **(C)** Dog No. 3 (administration of 4 foreign bodies). **(D)** Dog No. 4 (administration of 5 foreign bodies). **(E)** Dog No. 5 (administration of 2 foreign bodies).

The capsule endoscopic images of Group 2 are shown in Figure 5. The interpreters’ interpretation values for Group 2 are listed in Table 5. Interpreters 1–16 were veterinarians and interpreters 17–23 were veterinary students. There was no significant difference in the sensitivity between the veterinarian and veterinary student groups (veterinarian group: 98.7% vs. veterinary student group: 100%; *p* = 0.820) (Table 6).

**Table 5:** Interpretation values of interpreters in Group 2.

Number of dogs (number of FB)	Number of detected FB (detection rate for the number of FB administered)																						
	Veterinarian																Veterinary student						
	1	2	3	4	5	6	7	8	9	10	11	12	13	14	15	16	17	18	19	20	21	22	23
No. 1 (2)	2/2	2/2	2/2	2/2	2/2	2/2	2/2	2/2	2/2	2/2	2/2	2/2	2/2	2/2	2/2	1/2	2/2	2/2	2/2	2/2	2/2	2/2	2/2
No. 2 (3)	3/3	3/3	2/3	2/3	3/3	3/3	3/3	3/3	3/3	2/3	2/3	3/3	3/3	3/3	1/3	3/3	3/3	3/3	2/3	3/3	2/3	3/3	3/3
No. 3 (4)	3/4	3/4	4/4	2/4	4/4	3/4	3/4	3/4	4/4	2/4	4/4	3/4	3/4	3/4	2/4	3/4	3/4	3/4	2/4	3/4	4/4	3/4	3/4
No. 4 (5)	4/5	4/5	3/5	2/5	3/5	3/5	4/5	4/5	4/5	4/5	4/5	4/5	3/5	4/5	1/5	3/5	4/5	4/5	3/5	4/5	2/5	3/5	3/5
No. 5 (2)	1/2	2/2	1/2	2/2	2/2	1/2	2/2	2/2	2/2	1/2	2/2	1/2	2/2	1/2	0/2	1/2	2/2	1/2	1/2	2/2	2/2	2/2	2/2

FB, foreign body.

**Table 6:** Total number of interpretation values of interpreters in Group 2.

	Total number of interpretation values	
	Veterinarians (*n* = 16)	Veterinary students (*n* = 7)
Number of interpretation values which one or more FB were detected	79	35
Number of interpretation values which FB were not detected	1	0
Sensitivity	98.7%	100%

FB, foreign body.

### 3.3 Comparison of detection capability of capsule endoscopy based on the number of foreign bodies

A significant difference was observed in the detection capability of capsule endoscopy depending on the number of foreign bodies. The mean ± standard deviation (SD) of interpretation values were as follows: No. 1, 97.8 ± 10.4%; No. 2, 88.2 ± 19.3%; No. 3, 76.0 ± 15.9%; and No. 4, 66.9 ± 16.6%. These differences were statistically significant (*p* < 0.05).

### 3.4 Comparison of detection capability of capsule endoscopy based on the changes in gastric environment

A significant difference was observed in the detection capability of the capsule endoscopy depending on the state of the gastric environment. The mean ± SD of interpretation values were as follows: No. 1, 97.8 ± 10.4%, and No. 5, 78.2 ± 29.4%. These differences were statistically significant (*p* < 0.05).

### 3.5 Calculating the intra-rater reliability within veterinarians and veterinary students

We found that the ICC (95% CI) was 0.61 (0.33, 0.93) between veterinarians and 0.55 (0.21, 0.92) between veterinary students, indicating moderate reliability as shown in Table 5.

**Figure 6:**
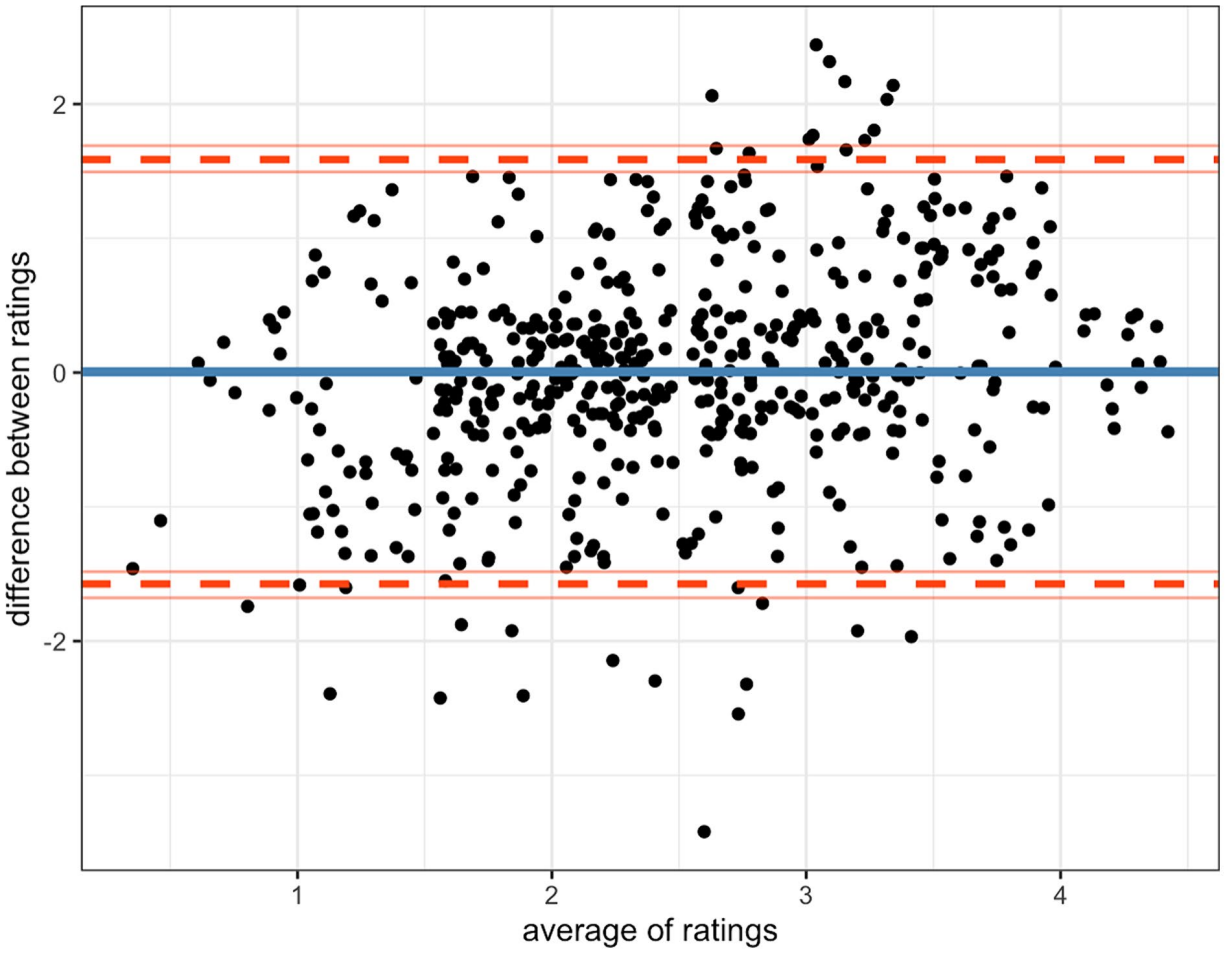
Bland–Altman plot result for estimating the agreement of ratings between the veterinarian group and the veterinary student group.

For the agreement between the two ratings, the Bland–Altman plot is shown in Figure 6. The mean difference was 0.005 with a standard deviation of 0.806. The upper limit of agreement was 1.586, and the lower limit was −1.574. We found that 93.7% of the ratings were within the limits of agreement.

## 4 Discussion

Various diagnostic tools can be used to detect foreign bodies in small animals when ingestion is suspected. Abdominal radiography and ultrasonography are diagnostic tools frequently used to detect gastrointestinal foreign bodies (21, 22). In two veterinary studies, abdominal radiography detected 56 and 69% of gastrointestinal foreign bodies (9/16 and 337/483, respectively), and ultrasonography detected 100% of gastrointestinal foreign bodies (16/16 and 123/123, respectively) (16, 20). Foreign bodies that are not detected on abdominal radiography can be detected using ultrasonography, which can also provide evidence of intestinal perforation. Therefore, abdominal ultrasonography is a more useful diagnostic tool than abdominal radiography and is recommended if only one diagnostic tool can be used (16). However, the detection of foreign bodies is limited when excessive gas is present in the gastrointestinal tract. Furthermore, surgeon’s experience plays an important role in the detection of foreign body with ultrasonography (16, 22, 23). Barium contrast radiography detected 86% of the gastrointestinal foreign bodies (20). However, it is contraindicated in patients with persistent vomiting or suspected intestinal perforation (22, 24). Gastrointestinal endoscopy or exploratory laparotomy allows simultaneous diagnosis and extraction of foreign bodies. However, these procedures cannot be performed if anesthesia is not indicated in patients with systemic illnesses. Therefore, it is not suitable as a screening tool.

This study evaluated the potential of capsule endoscopy as a screening tool for dogs with suspected foreign body ingestion. For this purpose, sensitivity was defined as the rate at which at least one foreign body was found, as the determination of the presence of any foreign body was considered imperative rather than measuring the exact number of foreign bodies. In this study, with the exception of one interpreter, all interpreters detected at least one foreign body in the administered group. Therefore, if at least one foreign body is detected, its removal is indicated. In the capsule-only group, 11 interpreters detected hair, bubbles, nylon tape, residual debris, and light from the capsule reflected by water present in the stomach, all of which affected the estimate of specificity.

Dogs may ingest more than one foreign body, and if multiple foreign bodies are present, they may vary in nature (18, 20). In this study, the foreign body detection capability of the capsule endoscopy was much lower when two or more gastric foreign bodies were present than when only one was present. However, because this study used foreign bodies of similar size and shape, future research should evaluate foreign bodies of a greater variety. Additionally, when foreign bodies are ingested, other materials, such as food, are rarely not ingested. If a substantial amount of ingesta is present in the stomach, detection of foreign bodies can be challenging using imaging evaluation. In this study, the detection capability of the capsule endoscopy for foreign bodies was significantly lower in cases where the stomach environment contained ingesta than when a foreign body was present in the stomach alone.

The interpretation capabilities of the veterinarians were compared with those of the veterinary students. The operator expertise plays an important role in radiography and ultrasonography. However, capsule endoscopy does not require any special manipulation techniques and allows direct detection of foreign bodies in real time. Students were capable of detecting foreign bodies in this study and were no less successful than veterinarians. This may be a major advantage of capsule endoscopy.

In this study, a hand-held magnet was used to manipulate the capsule movement, and foreign bodies were detected in real time through the receiver’s screen. Healthy human volunteers have been evaluated for esophageal and stomach health by magnetic assisted capsule endoscopy (MACE) using a hand-held external magnet, and no specific adverse effects were observed (25, 26). Recent studies evaluated the upper gastrointestinal tract using MACE. The safety, maneuverability, and visualization of MACE have been demonstrated, and the clinical value of this procedure is well-established (27–29). MACE allows for the movement and rotation of the capsule, allowing the operator to actively navigate to the desired view by directly manipulating the capsule. Therefore, it is assumed that the use of MACE in this study would have been even more helpful in detecting gastric foreign bodies.

Unlike conventional gastrointestinal endoscopy, capsule endoscopy does not allow the removal of gastric residues or distention of the gastric cavity. In human medicine, several studies have used MACE to detect landmarks in the stomach (27, 28, 30). In one study, participants ingested an air-producing powder and a large amount of water to distend the stomach before performing capsule endoscopy, which allowed for improved visualization of the anatomical landmarks of the stomach (28). In this study, only a small amount of water (5 mL) was administered to allow for ease of swallowing the capsule, and capsule endoscopy was performed without gastric distension. If a large amount of water or air-producing powder had been ingested to distend the stomach, better visualization of the gastric cavity could have been achieved. In patients suspected of ingesting a foreign body, it is crucial to evaluate the appropriateness of administering a large volume of water or air-generating powder. Additionally, the feasibility of performing capsule endoscopy without inducing adverse effects must be carefully considered.

To the best of our knowledge, there have been no reports of capsules passing through the pylorus in cats. It may be possible to detect gastric foreign bodies using capsule endoscopy in cats; however, the capsule itself may become a foreign body due to gastric retention. Therefore, capsule endoscopy is currently unsuitable as a screening tool for foreign bodies detection in cats. Previous research in dogs compared gastric transit time (GTT) and small bowel transit time (SBTT) using the currently existing capsule and a minimized capsule model (31). The GTT was reduced for the minimized capsule model compared with the existing capsule. However, since the GTT was compared only in beagle dogs, capsules may or may not pass through the pylorus of small dogs or cats without any gastric retention. Therefore, the usefulness of smaller capsules has not been evaluated in small dogs or cats, in which gastric retention of capsules is more likely to occur. This study included only beagle dogs and all capsules were excreted into the feces without gastric retention. Therefore, further studies should be conducted to evaluate the utility of capsule endoscopy for small dogs or cats.

This study had some limitations. The use of virtual foreign bodies in the form of food items may not completely replicate the challenges encountered when detecting foreign bodies ingested by dogs in clinical settings. However, these food items were selected to simulate foreign bodies that are typically encountered in dogs, such as chewing toys or other objects. Additionally, this study was performed using healthy beagles, which may not fully represent the spectrum of dog breeds and scenarios encountered in clinical practice.

Although our study confirmed capsule endoscopy as a promising diagnostic tool for detecting foreign bodies in the canine stomach, further research is needed to explore its utility in dogs of different breeds, ages, and clinical presentations. The detection capability of capsule endoscopy should also be evaluated in cases in which foreign bodies vary in size, shape, and composition. Future studies should investigate the detection capability of capsule endoscopy in different segments of the gastrointestinal tract and cases with varying gastrointestinal transit times.

In conclusion, this study proved that capsule endoscopy is a highly sensitive and specific diagnostic tool for detecting foreign bodies in the canine stomach. Capsule endoscopy may be a valuable alternative diagnostic approach for identifying foreign bodies in the stomach, particularly when conventional imaging or other invasive approaches are unsuitable or pose challenges. Further research is required to assess this in various breeds of dogs, other species, and different types of foreign bodies.

## Data availability statement

The raw data supporting the conclusions of this article will be made available by the authors, without undue reservation.

## Ethics statement

The animal study was approved by the Institutional Animal Care and Use Committee (IACUC) of Gyeongsang National University (GNU-190827-D0041). The study was conducted in accordance with the local legislation and institutional requirements.

## Author contributions

U-YK: Conceptualization, Data curation, Formal analysis, Investigation, Methodology, Software, Validation, Visualization, Writing – original draft. YK: Conceptualization, Data curation, Formal analysis, Methodology, Resources, Validation, Visualization, Writing – review & editing. JL: Data curation, Formal analysis, Validation, Visualization, Writing – review & editing. MK: Data curation, Investigation, Methodology, Validation, Visualization, Writing – review & editing. HJ: Data curation, Funding acquisition, Project administration, Software, Supervision, Writing – review & editing, Writing – original draft. D-IJ: Conceptualization, Formal analysis, Funding acquisition, Investigation, Methodology, Project administration, Resources, Software, Supervision, Visualization, Writing – review & editing, Writing – original draft.
